# Can eDNA Replace Trawl Surveys for Estuarine Species Distribution Modeling: Insights From *Collichthys lucidus* in the Yangtze River Estuary

**DOI:** 10.1002/ece3.71854

**Published:** 2025-08-21

**Authors:** Xiaoyu Geng, Wei Tang, Jianhui Wu, Chunxia Gao, Xuefang Wang

**Affiliations:** ^1^ College of Marine Living Resource Science and Management Shanghai Ocean University Shanghai China; ^2^ Shanghai Aquatic Wildlife Conservation and Research Center Shanghai China; ^3^ Joint Laboratory for Monitoring and Conservation of Aquatic Living Resources in the Yangtze Estuary Shanghai China

**Keywords:** critical habitat requirements, environmental DNA (eDNA), species distribution model, species occurrence, Yangtze river estuary

## Abstract

Species distribution data underpin species distribution models (SDMs), which are essential for identifying habitat preferences and informing conservation strategies. Environmental DNA (eDNA) has emerged as a powerful tool for aquatic biodiversity monitoring. However, its reliability in supporting SDMs—especially in dynamic estuarine systems—remains uncertain. To address this, this study evaluated the modeling performance of trawl‐derived and eDNA‐derived occurrence data for the benthic fish *Collichthys lucidus* , using paired surveys conducted in the Yangtze River Estuary in August and November 2021. Species distribution models were developed using both MaxEnt and ensemble modeling (EM) approaches. The results showed that although eDNA and trawl data produced similar performance metrics (AUC, Kappa, RMSE), eDNA‐based models exhibited weaker spatial discrimination and inconsistent seasonal predictions, misidentifying offshore areas as unsuitable under certain conditions. In contrast, trawl‐based models consistently identified suitable offshore habitats and highlighted salinity as the dominant driver, whereas eDNA models emphasized dissolved oxygen. These findings suggest that using eDNA as a sole input for spatially explicit habitat modeling in estuaries requires caution, particularly where precise location information is critical.

## Graphical abstract

We investigated the habitat suitability of *Collichthys lucidus* in the Yangtze River Estuary using both trawl surveys and environmental DNA (eDNA) technology. Trawl surveys indicated that *C. lucidus* primarily inhabits high‐salinity nearshore waters, while eDNA sampling revealed its presence in the freshwater of the river due to the transport of seawater intrusion. Our study emphasizes the importance of cautious evaluation when using eDNA to predict species distribution in dynamic estuarine environments.

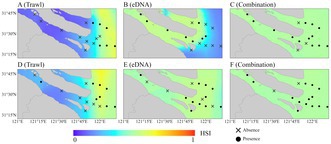

## 1 Introduction

Species distribution models (SDMs) are mathematical models that combine species distribution data with environmental data to predict past, present, or future species distributions (Elith and Leathwick 2009; Pecchi et al. 2019). As a common tool and model for ecological and biogeographic research (Peterson et al. 2015), SDMs have been widely applied to terrestrial, marine, and freshwater environments, including for protected area planning, climate change impact assessment, biodiversity evaluation, and species habitat modeling (Melo‐Merino et al. 2020).

Species occurrence data form the basis for SDMs and can be collected from literature, specimens, or the field (Melo‐Merino et al. 2020). The accuracy of the SDMs depends not only on the sampling effort (number of occurrence data) used to generate the model (Aizpurua et al. 2015), but also on the accuracy of occurrence data, which includes uncertainty in species identification, bias in the selection of sample locations, and incomplete spatial coverage of the true distribution of species (Kramer‐Schadt et al. 2013; Guisan et al. 2017). In traditional approaches for the modeling of marine organisms, the most common method of collecting species occurrence data is through surveys and monitoring using various gears (e.g., trawl, gillnet, trap etc.), which have the advantage of being real and reliable (Pennino et al. 2016), but have the disadvantage of being invasive, gear selectivity, labor‐intensive, time‐consuming, and expensive (Thomsen and Willerslev 2015), and hence, do not fully satisfy the needs of exploring the oceans. This situation calls for the development of new and emerging species monitoring methods to collect species occurrence data (Rondinini et al. 2006).

In recent years, environmental DNA (eDNA) technology has been widely applied in aquatic biodiversity monitoring due to its advantages of high sensitivity, biological friendliness, operational simplicity, and high efficiency (Aizpurua et al. 2015; Thomsen et al. 2012). Given these characteristics, eDNA has the potential to provide species occurrence data for habitat modeling and has already been put into practice in some cases. For example, He et al. (2023) used DNA information extracted from water samples to model the regional habitat suitability of *Acaudina molpadioides* in Qingchuan Bay. Shelton et al. (2022) combined traditional acoustic trawl surveys with eDNA sampling to develop spatial statistical models for analyzing large‐scale species distribution and abundance along the Pacific Coast, and both methods were found to provide valuable information.

However, there are also notable limitations in using eDNA to support habitat modeling, including a certain degree of spatial drift (Andruszkiewicz Allan et al. 2021) and the occurrence of false‐positive and false‐negative results (Jerde 2021). A relatively typical example is the fish survey conducted in the Jiangsu section of the Yangtze River, where eDNA detection identified 11 more fish species than traditional fishing gears. Nonetheless, these additional species are believed likely not to reflect the actual species distribution, but rather to result from shed tissues or excretions of fish carried by upstream water flow (Ding 2021). These uncertainties in species occurrence data may further lead to biased model outputs.

Estuaries, as open areas where oceans and rivers meet, provide spawning grounds, nurseries, and migratory corridors for many marine species, and play a critical role in fish life history processes and in shaping the spatial patterns of community compositio (McLusky and Elliott 2004). However, they are also characterized by high ecological heterogeneity and dynamic environmental conditions (Hibma et al. 2004). In temperate estuaries, fish richness continuously declines from the ocean toward the river (Selleslagh et al. 2009; Whitfield et al. 2012). Compared to freshwater species that spawn in estuaries and primarily inhabit riverine areas, marine and estuarine species dominate the high‐salinity regions near the ocean, resulting in pronounced spatial variation in fish communities (Nicolas et al. 2010). Therefore, accurate monitoring of fish distribution has significant practical applications in the management and conservation of estuarine ecosystems.

The Yangtze River Estuary is the largest estuary in the western Pacific Ocean and is characterized by a typical bifurcated geomorphology, where saline water from the north branch can intrude into the south branch, creating a pronounced salinity gradient across the region (Yuan et al. 2002; Zhuang 2006; Qiu et al. 2012). This unique hydrodynamic and spatial structure provides a natural experimental setting for simulating the downstream transport of waterborne materials from upstream areas and evaluating its ecological consequences.

*Collichthys lucidus* is a warm‐water, nearshore demersal fish commonly found in fish surveys of the Yangtze River Estuary. Its pronounced response to salinity variation (Zhang et al. 2010) makes it an ideal representative species for investigating distribution patterns and key habitat requirements in fragmented estuarine environments. In this study, we used *C. lucidus* occurrence data obtained from both traditional trawl surveys and eDNA sampling to construct habitat suitability models based on presence‐only and ensemble modeling approaches. The aim is to assess whether eDNA can effectively replace or complement conventional survey data in hydrodynamically complex environments, providing empirical evidence to advance the understanding of the potential and applicability of eDNA for species distribution modeling.

## 2 Materials and Methods

### 2.1 Study Area Overview

**FIGURE 1:**
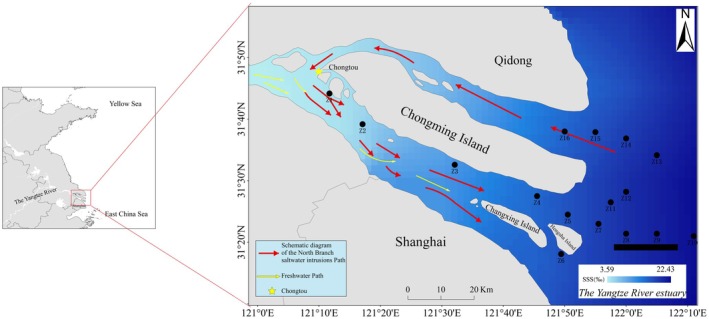
Distribution map of fish monitoring and survey stations in the Yangtze River Estuary in 2021 (the red arrows in the figure represent the path of saltwater intrusion in the north branch of the estuary, and the yellow arrows represent the river path map).

The Yangtze River Estuary, located at the confluence of freshwater from the Yangtze River and saline water from the East China Sea, is a typical multi‐distributary estuary characterized by a complex bifurcated morphology with parallel southern and northern branches (Dai et al. 2013; Qiu et al. 2012). The south branch is primarily influenced by river discharge, with substantial freshwater input throughout the year and relatively low salinity. In contrast, the north branch is shallower, strongly affected by tidal forces, and frequently experiences seawater intrusion, resulting in significantly higher salinity than the south branch (Song et al. 2020). Under conditions of reduced river runoff, astronomical high tides, or strong tidal dynamics, high‐salinity seawater can advance westward along the north branch and intrude into the south branch via tributaries such as the Hengsha Channel, leading to a saltwater intrusion phenomenon (Ge et al. 2022). The bifurcated estuarine structure and the saltwater intrusion pathway via the north branch are shown in Figure 1.

### 2.2 Collection of Occurrence Data

Two trips of bottom‐trawl surveys combined with eDNA sampling were conducted during August and November 2021 to evaluate aquatic resources in the Yangtze River Estuary under Shanghai's jurisdiction (121.4°E–122.2°E, 31.3°N–31.7°N). A total of 16 fixed stations were set up for monitoring fishery resources and the environment. The distribution of the stations is shown in Figure 1.

#### 2.2.1 Data From Bottom‐Trawl Surveys

The bottom beam trawl used for sampling had a mouth width of 6 m, cod‐end mesh size of 2 cm, and height of 2 m. One tow was performed at each station at a speed of 2 NM/hour for 30 min. The presence or absence of *C. lucidus* at each station was determined based on catch data, and these occurrence records were subsequently used in both the MaxEnt and ensemble modeling (EM).

#### 2.2.2 Data From eDNA Surveys

The water depth at sampling stations ranged from 1.4 to 16.9 m. At each station, surface, middle, and bottom water samples were separately collected using a water sampler and then thoroughly mixed. A 1000 mL aliquot of the composite sample was filtered on‐site using 47 mm diameter, 0.45 μm pore‐size mixed‐fiber membranes under vacuum. Filtration equipment was pre‐sterilized in 75% ethanol. The filtered samples were then stored at −80°C in 1.5 mL brown centrifuge tubes and promptly transported to the laboratory. Filtration blanks and negative controls were coextracted alongside the samples and were subjected to the same protocol as the samples. DNA extraction was carried out using the DNeasy Blood and Tissue kit, following the protocols of Ficetola et al. (2008), Dejean et al. (2011), and Renshaw et al. (2015). Filter membranes were cut, ground, and soaked in 300 μL of buffer ATL and 20 μL of proteinase K. Incubation at 56°C for 1.5 h was followed by kit instructions, including washing and elution in 40 μL of AE buffer. Extracted eDNA concentration was immediately determined using a Nanodrop 2000 and assessed on a 1.0% agarose gel, showing no data or bands for filtration blanks or negative controls.

PCR amplification utilized FISH eDNA universal primers (Mifish‐F [5′‐GTCGGTAAAACTCGTGCCAGC‐3′] and Mifish‐R [3′‐GTTTGACCCTAATCTATGGGGGGTGATAC‐5′]), generating fragments of 297 ± 25 bp. The amplified fragment is located within a specific region of the mitochondrial COI gene and contains abundant species‐specific variation sites. Previous studies have demonstrated that this fragment length is effective for detecting species within the Sciaenidae family, including *C. lucidus* (Miya et al. 2015), and it has also been successfully used to detect *C. lucidus* in fish community monitoring in the Yangtze River Estuary (Wang et al. 2023). Three PCR replicates were performed for each sample, with environmental samples, filtration blanks, and negative controls included. The 25‐μL amplification system contained 5× reaction buffer (5 μL), 5 μL of 5× GC buffer, 2 μL dNTP (2.5 mmol/L), 1 μL each of forward and reverse primers (10 μmol/L), 2 μL template DNA (20 ng/μL), 8.75 μL double‐distilled water, and 0.25 μL Q5 DNA polymerase. A two‐step PCR protocol involved 95°C pre‐denaturation for 5 min, 55 cycles of denaturation at 95°C for 30 s and annealing at 60°C for 30 s. Gel electrophoresis (1.5% agarose gel) confirmed no amplification in filtration blanks or negative controls. Sequencing on the Illumina MiSeq platform completed the process.

Raw sequencing data were saved in FASTQ format and initially screened based on sequence quality. The raw bipartite sequencing data were spliced and deduplicated using Vsearch (v2.13.4_linux_x86_64) and cutadapt (v2.3) software and clustered into operational taxonomic units (OTUs) according to a 97% similarity level to obtain the representative sequences and OTU tables. Representative OTU sequences were compared with the reference sequence databases NCBI (https://www.ncbi.nlm.nih.gov/) and MitoFish (http://mitofish.aori.u‐tokyo.ac.jp/download.html) for taxonomic annotation of crop species, and site distribution information on the occurrence of *C. lucidus* was recorded and organized.

### 2.3 Acquisition and Preprocessing of Predictor Variables

The environmental variables were selected based on previous characteristic studies of fish ecological habitats in the Yangtze River Estuary (Pan et al. 2021), including temperature (T), salinity (S), dissolved oxygen (DO), pH, and chlorophyll concentration (Chl) were measured. All marine environmental data were downloaded from the COPERNICUS website (http://marine.copernicus.eu/). We used Pearson's correlation coefficients (*r*), using an |*r*| > 0.7 to cull collinear predictors (Dormann et al. 2013). The retained environmental variables were coupled with species occurrence data, and the specific range and resolution of each environmental variable are shown in Table 1. ArcMap 10.4 software was used to convert the netCDF raw files into raster layers in the TIFF format. Due to the small spatial extent of this study area, raster layers were converted to ASCII format files with a spatial resolution of 0.083° × 0.083° and identical boundaries.

**TABLE 1:** Overview of environmental variables used for habitat modeling in this study.

Variable name	Abbreviation	Realm	Unit	Spatial resolution
Temperature	T	16–30	°C	0.25° × 0.25°
Salinity	S	0–17	‰	0.25° × 0.25°
pH	pH	7–8	—	0.25° × 0.25°
Dissolved oxygen	DO	243–332	mmol·m^−3^	0.25° × 0.25°
Chlorophyll concentration	Chl	6–14	mg·m^−3^	0.25° × 0.25°

### 2.4 Modeling, Prediction, and Evaluation

To enhance the stability and reliability of predictions, this study employed two modeling approaches for comparison: the Maximum Entropy model (MaxEnt) and Ensemble Modeling (EM). MaxEnt, based on the principle of maximum entropy, constructs species distribution models using presence data and their relationships with environmental variables, and is known for its high predictive accuracy (Phillips and Dudík 2008). EM, on the other hand, integrates both presence and absence data and combines predictions from multiple individual models following specific rules to produce a more robust composite model (Araújo and New 2007). This approach effectively reduces uncertainty caused by model overfitting (Dormann et al. 2018; Kaky et al. 2020) and improves overall prediction performance.

#### 2.4.1 MaxEnt Modeling

The predictive results of the MaxEnt are influenced by the type of features and the settings of the regularization multiplier (Galante et al. 2018). To improve model performance, this study combines different feature classes and the regularization multiplier during the modeling process. The feature classes include linear (l), product (p), quadratic (q), and hinge (h), as well as combinations thereof. The regularization multiplier was increased in increments of 0.1 within the range of 0.1–4. Selecting sampling sites where the target species was not captured in the survey data as pseudo‐missing points helps reduce bias caused by arbitrary selection of background points (Barbet‐Massin et al. 2012). The Akaike Information Criterion (AICc, Akaike (1970)) was used to select the optimal model, as AICc is particularly suitable for small sample datasets (Hurvich and Tsai 1989). After obtaining the optimal model, habitat predictions are made in conjunction with environmental variables, and the model is run 100 times to reduce random error. The detailed parameters of the optimal model are listed in Table A1. The model is constructed using the “maxnet” package in R (version 4.4.2) (Phillips et al. 2017).

#### 2.4.2 EM Modeling

In this study, six commonly used models were selected as base models for ensemble modeling: Generalized Linear Model (GLM), Generalized Additive Model (GAM), Flexible Discriminant Analysis (FDA), Random Forest (RF), Artificial Neural Network (ANN), and MaxEnt (using the “maxnet” package). A five‐fold cross‐validation (*K* = 5) was applied to improve model generalization (Hyndman and Athanasopoulos 2018), and each model was run 100 times to reduce randomness. Ensemble modeling was conducted by selecting individual models with high predictive accuracy (AUC > 0.7) for inclusion (Hao et al. 2019). A weighted mean ensemble approach was then applied to combine predictions, reflecting the overall trend of model outputs (Hao et al. 2020). Parameter settings for all base models are listed in Table A2. All modeling procedures were implemented in R (version 4.4.2) using the “biomod2” package (Thuiller et al. 2009).

#### 2.4.3 Model Evaluation

To minimize potential bias introduced by relying on a single evaluation metric, this study employed three commonly used indicators to comprehensively assess model performance: (1) Area Under the Receiver Operating Characteristic Curve (AUC), which measures the model's discriminatory ability. Values range from 0 to 1, with values closer to 1 indicating better predictive performance. Generally, models with AUC > 0.7 are considered to have good predictive ability (Elith et al. 2006). Although AUC has been debated in species distribution modeling, it remains useful when true absence data are available (Lobo et al. 2008; Jiménez‐Valverde 2012); (2) The Kappa coefficient, which evaluates model consistency, is widely used for accuracy assessment in presence–absence models. Values range from 0 to 1, with higher values indicating stronger agreement between predictions and observations (Howard et al. 2014); (3) Root Mean Square Error (RMSE), which reflects the average deviation between predicted and observed values. Lower RMSE values indicate higher predictive accuracy (Thibaud et al. 2014).

#### 2.4.4 Importance of Environmental Variables in Habitat Suitability

In the MaxEnt model, permutation importance is used to quantify the contribution of environmental variables. This method randomly permutes the values of a given environmental variable, reevaluates the model performance, and assesses the importance of the variable based on the change in the model's AUC; a greater decrease in AUC indicates a higher importance of that variable to the model (Phillips and Dudík 2008). For EM, the permutation test method implemented in the biomod2 package is used to calculate the importance values of variables, and the final importance assessment is obtained by integrating the results of different individual models through a weighted average (Naimi and Araújo 2016).

### 2.5 Model Output and Visualization

The predicted habitat suitability results of *C. lucidus* from each model were exported in ASCII format. Model evaluation metrics, environmental variable importance, and response curve data were exported as CSV files. These data were visualized using ArcMap 10.4 and Origin 2022 software.

## 3 Results

### 3.1 Species Distribution Obtained From Different Sources of Species Occurrence Data

**FIGURE 2:**
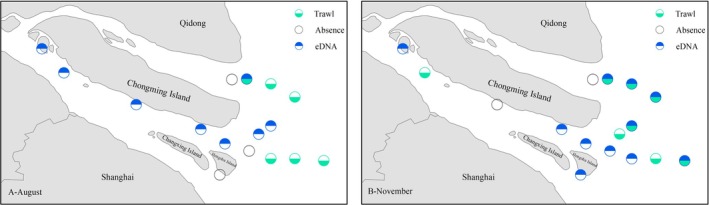
Schematic diagram of stations with eDNA and trawl detections of *Collichthys lucidus* in the Yangtze River Estuary for August and November 2021. (A) Distribution of stations detected during the August survey. (B) Distribution of stations detected during the November survey.

During the August 2021 survey, there was a significant difference in the spatial distribution of occurrence sites of *C. lucidus* , as indicated by the two methods. The eDNA technique revealed that *C. lucidus* was detected at eight sites, or 50% of the total, but was almost exclusively distributed in the waters of the south branch of the Yangtze River Estuary, whereas the trawl collected the target species at six sites (37.5% of the total), which were all distributed in nearshore marine areas outside estuaries (Figure 2A). Only one site (Z15) showed targets identified using both methods (Figure 2A).

The survey conducted during November 2021 showed that trawl‐collected *C. lucidus* sites remained predominantly outside the estuary, with 11 eDNA detections (68.7%). However, several sites in the entrance area of the south branch continued to have only eDNA indicative of *C. lucidus* (Z4, Z5, and Z6, Figure 2B), whereas multiple detections in the nearshore marine areas outside estuaries appeared to have targets investigated by both methods (Z13, Z14, Z15and Z12, Figure 2B).

### 3.2 Comparison of Model Performance Across Different Modeling Methods

**FIGURE 3:**
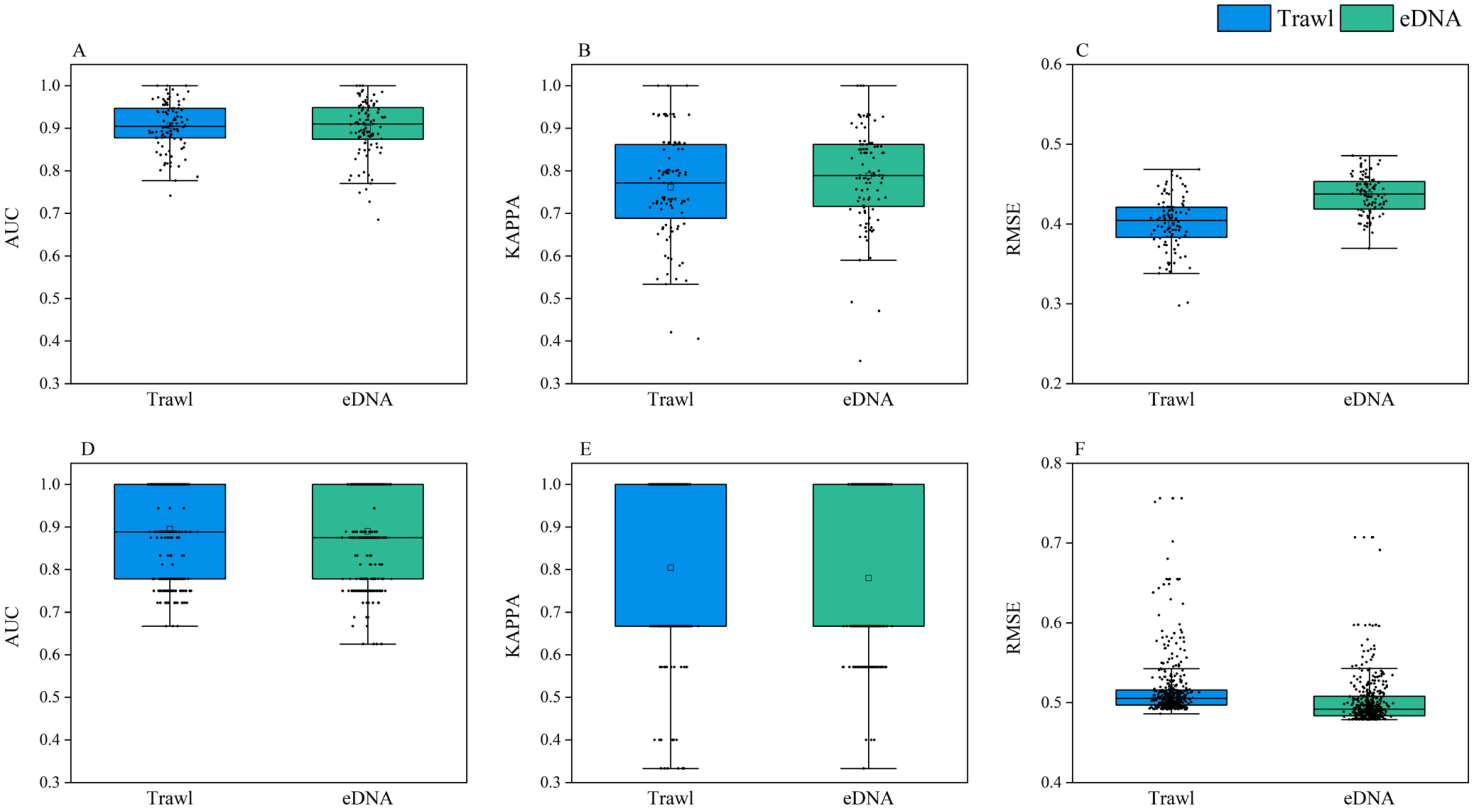
Comparison of performance evaluation metrics for species distribution models (SDMs) of *Collichthys lucidus* in the Yangtze River Estuary based on MaxEnt and Ensemble Modeling. (A–C) Results from MaxEnt models; (D–F) Results from ensemble models.

In terms of AUC and Kappa values, the overall performance of both the MaxEnt and EM constructed using either trawl or eDNA data was generally comparable (Figure 3A,B,D,E). However, the RMSE comparisons showed opposite trends between the two modeling approaches: for the MaxEnt model, the RMSE median based on eDNA data was higher than that based on trawl data (0.437 vs. 0.401, Figure 3C), whereas in the EM, the RMSE for eDNA data was slightly lower than that for trawl data (0.504 vs. 0.519, Figure 3F). Therefore, it is difficult to conclusively determine which type of species occurrence data is more suitable for modeling solely based on model performance metrics.

### 3.3 Comparison of the Spatial Structure of Predicted Habitat Suitability

**FIGURE 4:**
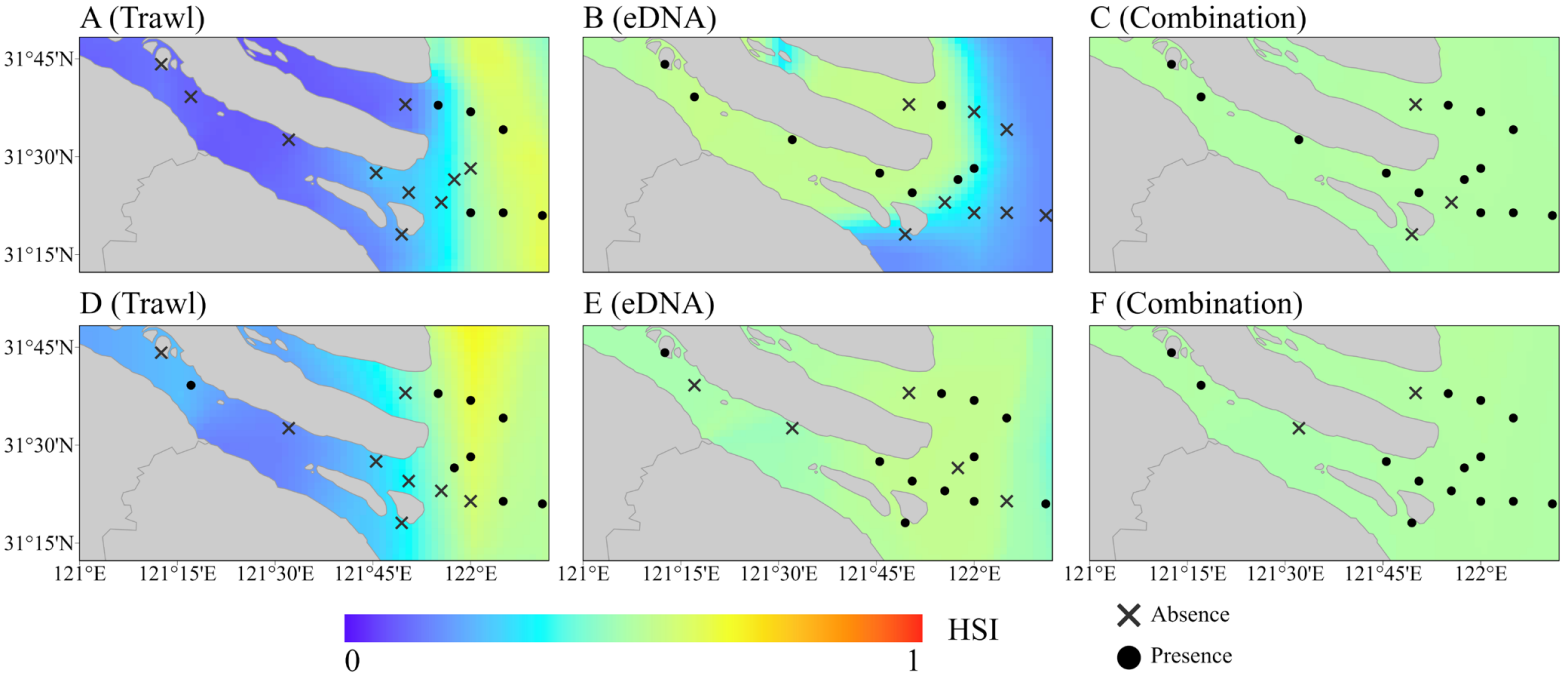
Predicted habitat suitability for *Collichthys lucidus* in the Yangtze River Estuary based on MaxEnt for August and November 2021. (A–C) Predictions for August; (D–F) predictions for November.

The ability of models to predict spatial structure—particularly their accuracy in distinguishing between freshwater and marine habitat suitability—is a key indicator of the reliability of species occurrence data. In the predictions generated by MaxEnt, those based on trawl survey data consistently distinguished well between areas of *C. lucidus* presence and absence across different months, correctly identifying the offshore waters outside the estuary as suitable habitats (Figure 4A,D). In contrast, the models built using eDNA data only effectively differentiated between freshwater and marine habitats in August, but misidentified offshore marine waters as unsuitable (Figure 4B). The results from November showed no clear spatial separation (Figure 4E). Additionally, the models that combined both data sources failed to clearly define habitat boundaries (Figure 4C,F).

**FIGURE 5:**
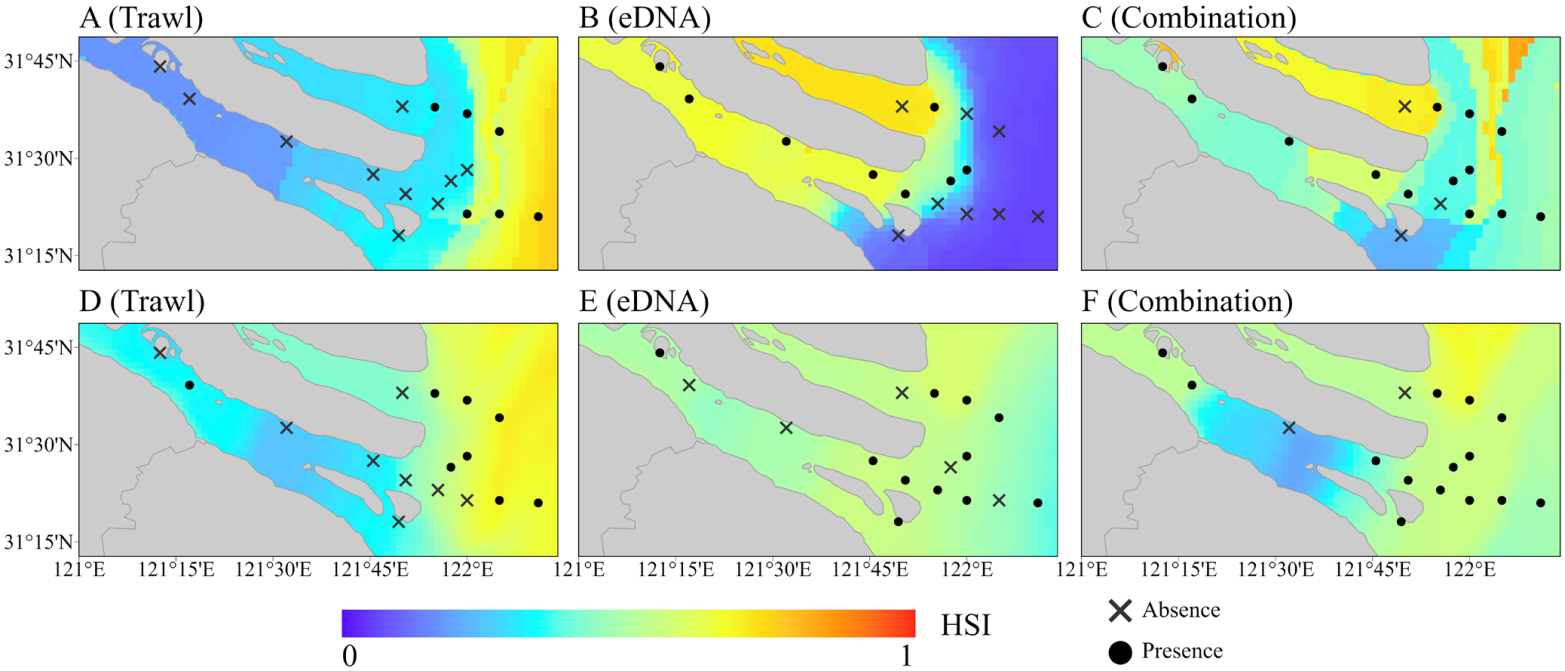
Predicted habitat suitability for *Collichthys lucidus* in the Yangtze River Estuary based on ensemble modeling (EM) for August and November 2021. (A–C) Predictions for August; (D–F) predictions for November.

The predictions from the EM exhibited a similar trend. However, the contrast between suitable and unsuitable habitats became more pronounced, further reinforcing the comparison of spatial prediction capabilities among different data types (Figure 5A–F).

### 3.4 Comparison of Key Habitat Requirements

**FIGURE 6:**
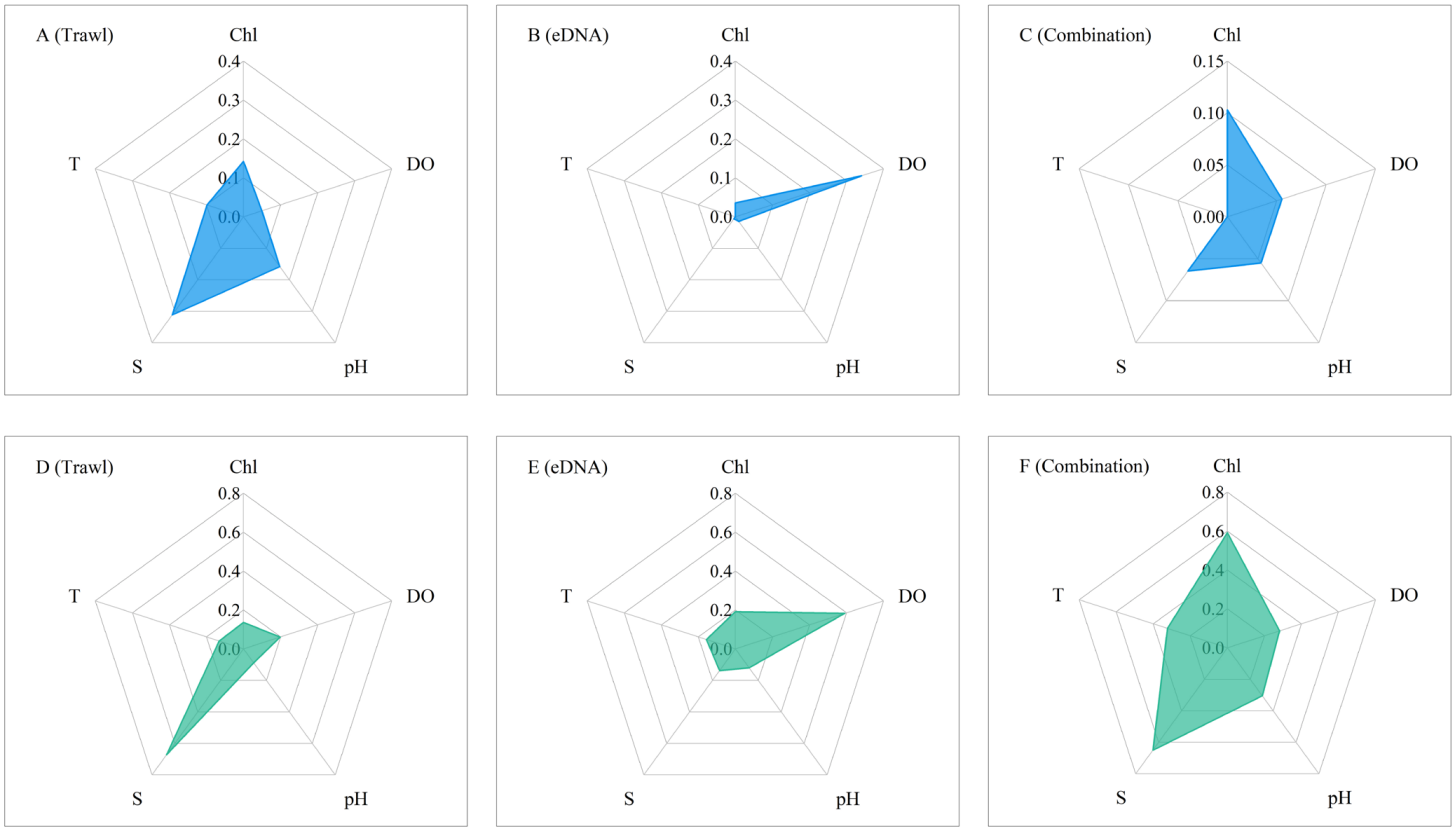
Relative importance of environmental variables for *Collichthys lucidus* based on permutation importance in MaxEnt and ensemble modeling (EM). (A–C) MaxEnt results; (D–F) EM results.

Models constructed using different species occurrence datasets exhibited notable differences in identifying key habitat variables. In models based on trawl data, both the MaxEnt and EM consistently identified salinity as the most important factor, far surpassing other environmental variables (Figure 6A,D). In contrast, models based on eDNA data highlighted dissolved oxygen as the dominant factor influencing habitat suitability, with salinity playing a less critical role in determining the distribution of *C. lucidus* (Figure 6B,E). In models combining both datasets, the importance of environmental factors appeared more balanced, with no single variable emerging as dominant (Figure 6C,F).

### 3.5 Environmental Variable Response Curve

**FIGURE 7:**
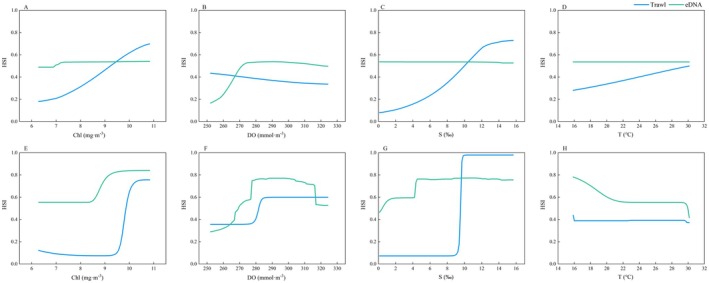
Response curves of *Collichthys lucidus* in the Yangtze River Estuary to various environmental variables, obtained using MaxEnt and ensemble modeling (EM). (A–D) MaxEnt results; (E–H) EM results. Chl, chlorophyll concentration; DO, dissolved oxygen; S, salinity; T, temperature.

The models constructed using different species occurrence datasets exhibited complex patterns of environmental partial dependence, with the most notable contrast observed between chlorophyll concentration and salinity. For Chl, both models based on trawl data and eDNA data consistently indicated an increasing trend in the HSI of *C. lucidus* with rising Chl levels (Figure 7A,E). However, in terms of salinity, the trawl‐based model showed a clear positive relationship, with HSI increasing significantly as salinity rose, whereas the eDNA‐based model exhibited no such trend—or even an opposite pattern (Figure 7C,G).

## 4 Discussion

### 4.1 Can eDNA Technology Provide Data for Modeling Species Distribution?

Theoretically, eDNA technology can provide three types of data for species surveys: occurrence, abundance, and biological habitat data (Pennino et al. 2016). Occurrence data refer to the presence or absence of a species as determined by detected DNA and can be used to assess biodiversity and monitor species distribution (Wu et al. 2023). When obtaining species presence data, both traditional gear and eDNA technology have errors, but their mechanisms are different. In the case of correct species identification, species information captured by nets must be real (Renshaw et al. 2015), but if the amount of effort is insufficient or based on the selectivity of gear, the results of the gear survey can produce false negatives (Green and Young 1993), that is, the fact that the location where the target species is not caught by gears does not necessarily imply that it does not occur in the sampling station. In contrast, although eDNA technology can provide more information on the occurrence of species at the sampling site (Doi et al. 2017), owing to experimental errors, unknown identification of hybridized species DNA, and missing genetic banks, the information provided about the presence of species is not completely accurate. If samples are not properly preserved, eDNA technology cannot be used to detect all species information (Wang et al. 2023).

In SDMs, catch per unit effort (CPUE) derived from traditional gear is often used as a proxy for species abundance (Yang et al. 2021). In eDNA studies, abundance is inferred from DNA concentration as a proxy for organismal biomass or density (Haxton and Friday 2020). However, the quantitative relationship between eDNA concentration and true abundance or biomass remains unclear and may vary across taxa and environments (Mariani et al. 2019). This uncertainty can reduce the suitability of eDNA concentration as an input for SDMs.

In this study, we considered species occurrence data derived from gear surveys—supported by physical specimens and standardized sampling effort—to be more reliable. These data were used to construct both presence‐only MaxEnt models and presence/absence‐based ensemble models. This dual modeling approach provides a comparative baseline for assessing the applicability of eDNA‐based distribution models under controlled conditions.

### 4.2 What Causes the Variation in Distribution Patterns of *C. lucidus* Indicated by eDNA and Trawl Data?

In recent years, eDNA technology has been widely used in the construction of species diversity indicators. Many of these surveys believe that eDNA technology can capture biological information in the region more comprehensively than traditional technology (Elith et al. 2011). Therefore, eDNA technology is considered a potential biodiversity monitoring tool (He et al. 2023). For example, Zhou et al. (2022) showed that eDNA identified 38.2% more fish species than bottom‐trawl surveys in the Zhoushan Sea. Zou et al. (2020) showed that eDNA metabarcoding identified 32.05% more fish species than bottom‐trawl surveys in the Pearl River Estuary. Gibson et al. (2023) found that eDNA detected higher species richness per 30 samples compared to bottom trawls or fyke nets. However, the assessment of species diversity within a region need not consider the precise matching of species occurrence information with the spatial location at each site and only needs a survey of the total region of interest and interpret results accordingly (DeAngelis and Yurek 2017). In contrast, SDMs are typically spatially explicit models in which the precise spatial location of species occurrence data is a prerequisite for matching and predicting habitat environmental requirements (Bertolino et al. 2020).

A comparison of the distribution (species occurrence points) of *C. lucidus* in the Yangtze River Estuary revealed significant differences between trawl and eDNA surveys (Figure 2). The most important difference was that the eDNA survey suggested that *C. lucidus* could be found widely in the freshwater of the river (i.e., the river‐dominated south branch of the Yangtze River Estuary, Figure 2), whereas trawl‐based observations indicated that, except for the Z3 site in November, the species almost exclusively inhabited nearshore waters outside the estuary, where the salinity was higher (Figure 2). Whereas Z3 is where the north saltwater intrusion meets the freshwater of the river, this site has a higher salinity than the other sites in the river and may be the edge of the distribution of *C. lucidus* in the river. Clearly, the conclusions of the trawl survey are consistent with the current knowledge, as *C. lucidus* is considered a benthic shallow marine fish (Zhang et al. 2010). In their historical survey of the Yangtze River Estuary, Zhang and Zhang (1985) used a stow net to continuously sample the south and north branches of the Yangtze River Estuary for 9 months from 1982 to 1983 and found that none of the stations in the south branch caught *C. lucidus* in stark contrast to the monthly collection of samples from the north branch of the Yangtze River Estuary, which experiences seawater intrusion (Figure A1).

In temperate regions, salinity influences fish distribution through physiological salinity tolerance, with spatial variations in salinity shaping fish community composition and causing significant spatial shifts along entire estuaries (Whitfield et al. 2012; Gibson et al. 2023). Studies on five estuaries in Japan have shown that the proportion of detected marine species increases with rising salinity, compared to freshwater and brackish species (Ahn et al. 2020). The difference in the distribution of *C. lucidus* between the north branch and south branch of the Yangtze River Estuary may be closely related to the distribution of salinity. The Yangtze River Estuary has three bifurcations and four outlets to the sea (Qiu et al. 2012; Figure 1), and its water quality is affected by both the freshwater transported by the Yangtze River and the saline water injected into the Yellow Sea, with a huge difference in the salinities of the south branch and north branch (Song et al. 2020). This phenomenon is attributed to the distinctive mechanism wherein the south branch, serving as a conduit for the Yangtze River's discharge into the sea, exhibits lower salinity. The substantial saltwater intrusion from tides, predominantly in the north branch, results in a notable influx of saltwater into the south branch, particularly at Chongtou (Figure 1). Consequently, the salinity level in the north branch of the Yangtze River Estuary becomes comparable to that of the outer sea. Thus, *C. lucidus* , a shallow‐sea fish, primarily inhabits the north branch and adjacent coastal waters (Zhang et al. 2010). However, owing to the transport mechanism of seawater intrusion in the Yangtze River Estuary, eDNA (or carcass) can be transported over long distances with the water flow (Deiner and Altermatt 2014), resulting in the frequent detection of eDNA signals from marine species in the lower‐salinity south branch. This leads to an apparent presence of *C. lucidus* in ecologically unsuitable habitats, which contradicts the patterns observed in trawl survey data.

### 4.3 What Is the Impact of Spatially Biased Species Occurrence Information on Estimates of Habitat Suitability and Environmental Requirements?

To assess the habitat suitability of *C. lucidus* , we applied SDMs based on three different occurrence datasets: trawl, eDNA, and a combined dataset. We compared the spatial characteristics of high‐suitability areas predicted by these models and found that those based solely on eDNA or the combined data identified the south branch of the Yangtze River Estuary as a key suitable area. This considerably expanded the predicted suitable range for *C. lucidus* in the estuary—an outcome inconsistent with known habitat preferences of this species (Figures 4 and 5).

Accurate occurrence information directly affects the outputs of SDMs in terms of areas of habitat suitability (Melo‐Merino et al. 2020). Traditionally, occurrence data have often included significant geographic bias, which arises mainly from inadequate sampling; for example, littoral areas are more likely to be surveyed than others because of their proximity or low‐cost accessibility, whereas offshore areas are difficult to sample or have insufficient data, resulting in missed or underestimated information on species occurrences (Araújo and Guisan 2006), making it impossible to identify some of the areas of habitat suitability. Estuaries, as open systems, may have eDNA monitoring influenced by external systems. Their highly dynamic environments and fluid nature complicate the spatial distribution of eDNA (Dickie et al. 2018). In some cases, the geographic range over which species information exists expands, resulting in spatially biased sample data, which results in inaccurate information on species presence in some areas (Yackulic et al. 2013).

Similarly, spatially biased sample data can lead to the misestimation of environmental requirements. Theoretically, suitable environmental conditions are a prerequisite for species occurrence. In addition, environmental information on the location of the species reflects the preference of the species for its habitat (Phillips et al. 2009). However, spatial bias in eDNA can cause models to fit environmental requirements associated with a particular geographic location incorrectly or excessively, resulting in unreliable models, thereby affecting model evaluation (Yackulic et al. 2013). Specifically, for the Yangtze River Estuary, the watershed of the estuarine‐dominated south branch and the marine‐dominated outer edge of the estuary have significant salinity differences; therefore, the distribution and frequency of occurrences in different regions will directly affect the judgment of critical environmental requirements for the species.

On the other hand, the level of salinity can also affect the accuracy of eDNA detection results. Tzafesta and Shokri (2025) found that eDNA detection rates in marine ecosystems are lower than those in river ecosystems; some studies indicate that eDNA degradation rates in seawater environments are generally faster than in freshwater (Thomsen et al. 2012; Sassoubre et al. 2016); additionally, Díaz‐Ferguson and Moyer (2014) found that high salinity may affect PCR results, reducing eDNA detection rates. This means that in low‐salinity areas, it is easier to detect the presence of target species using eDNA. These factors may further lead to models based on eDNA underestimating the role of salinity as a key habitat factor for marine fish.

## 5 Conclusions and Outlook

In the Yangtze River Estuary and adjacent coastal waters, species distribution modeling based on traditional gear‐derived data has been widely applied (e.g., Zhang et al. (2020); Pan et al. (2021); Xu et al. (2023); Wang et al. (2024)), yet no studies have evaluated the potential of eDNA‐derived occurrence data for such modeling efforts in this region.

This study explores the potential of using eDNA to provide species occurrence data for modeling marine fish distributions in estuarine areas. The findings on *C. lucidus* indicate that while eDNA can detect more occurrence points compared to trawling, it may not accurately match the spatial locations of species occurrences. Spatial accuracy has significant implications for SDMs in identifying primary suitable habitats and critical environmental requirements. In previous studies, despite being influenced by persistence and current transport, eDNA metabarcoding has successfully detected variations in fish communities at different spatial scales (< 1 km) within estuaries and between adjacent environments (Cole et al. 2022; Sellers et al. 2018). However, the spatial accuracy of species occurrence information provided by this technique may still be insufficient to meet the fine spatial scale requirements for habitat modeling in small areas. Thus, when sampling eDNA in a field with complex terrain, it is necessary to analyze hydrodynamic characteristics such as curvature, flow velocity, and topography and environmental factors such as ultraviolet light, temperature, and pH at the sampling points to determine their effects on eDNA stability and fluidity.

Due to the limited temporal coverage, most other fish species in our dataset were recorded too infrequently to enable robust modeling. As such, this study focused on *C. lucidus* as a representative case. To generate more generalizable conclusions about the use of eDNA for SDMs, future studies should include a broader suite of target species and extend sampling across multiple seasons. Such efforts will be critical for validating the ecological relevance and predictive performance of eDNA‐based distribution models in estuarine ecosystems.

## Author Contributions

**Xiaoyu Geng:** conceptualization (equal), methodology (equal), visualization (equal), writing – original draft (equal). **Wei Tang:** conceptualization (equal), methodology (equal), visualization (equal), writing – original draft (equal). **Jianhui Wu:** funding acquisition (equal), investigation (equal), project administration (equal). **Chunxia Gao:** project administration (equal), supervision (equal). **Xuefang Wang:** conceptualization (equal), formal analysis (equal), supervision (equal), writing – review and editing (equal).

## Conflicts of Interest

The authors declare no conflicts of interest.

## Supporting information


**Figure A1.** Distribution of *Collichthys lucidus* in the waters of the north and south branches of the Yangtze River Estuary based on stow net and stake‐hold net surveys during 1982–1983 (based on the results of Zhang and Zhang (1985)).
**Table A1**. Optimal MaxEnt model results based on modeling using different data sources for the *Collichthys lucidus* .
**Table A2**. Parameter settings for each individual modeling algorithm used in this study.

## Data Availability

The original species data and environmental data used in the MaxEnt model are included in the Supporting Information. All marine environmental data could be found from the COPERNICUS website (http://marine.copernicus.eu/). For further inquiries, the corresponding author may be contacted.
