# Supplementary material for: Can eDNA Replace Trawl Surveys for Estuarine Species Distribution Modeling: Insights From Collichthys lucidus in the Yangtze River Estuary
Source: Ecol Evol. 2025 Aug 21;15(8):e71854. doi: 10.1002/ece3.71854 (PMC12371124; doi:10.1002/ece3.71854)
Supplement: Supplementary file 1 — Figure A1. Distribution of Collichthys lucidus in the waters of the north and south branches of the Yangtze River Estuary based on stow net and stake‐hold net surveys during 1982–1983 (based on the results of Zhang and Zhang (1985)). Table A1. Optimal MaxEnt model results based on modeling using different data sources for the Collichthys lucidus . Table A2. Parameter settings for each individual modeling algorithm used in this study. [file ECE3-15-e71854-s001.docx]

Table A1 Optimal MaxEnt model results based on modeling using different data sources for the *Collichthys lucidus*

| Data type | Best features classes | Best regularization multiplier | AICc value |
| --- | --- | --- | --- |
| Trawl | lp | 0.1 | 38.96 |
| eDNA | qh | 1.5 | 38.17 |
| Combination | l | 0.8 | 41.59 |

Note: linear(l)、product(p)、quadratic(q) and hinge(h).

Table A2 Parameter settings for each individual modeling algorithm used in this study

| Method | Parameter | Value | Description |
| --- | --- | --- | --- |
| GLM | type | Quadratic | Type of GLM to be fitted |
|  | interaction.level | 0 | Level of interaction included in the model |
|  | test | AIC | Criteria for selecting the best-fitting model |
|  | mustart | 0.5 | Default starting value |
| GAM | algo | GAM_mgcv | Algorithms and optimization methods used |
|  | type | s_smoother | Smoothing type |
|  | k | −1 | Order of the smoother |
|  | Interaction.level | 0 | Level of interaction included in the model |
| FDA | method | mars | FDA modeling approach |
|  | degree | 2 | The highest order of interaction terms allowed in the model |
|  | nprune | 10 | The number of spline functions retained in the model |
| RF | ntree | 500 | Number of trees to be built |
|  | mtry | 2 | Number of variables to be tried at each split |
|  | nodesize | 5 | Minimum number of observations required in each terminal node (leaf) |
|  | maxnodes | NULL | Maximum number of terminal nodes allowed in the tree |
| ANN | size | NULL | Number of neurons in the hidden layer |
|  | decay | NULL | Weight decay parameter used to prevent overfitting |
|  | rang | 0.1 | Range of initial random weights |
|  | maxit | 200 | Maximum number of iterations for training the neural network |
| MaxEnt | regmult | 1 | regularization multiplier |

**Note:** GLM = generalized linear model; GAM = generalized additive model; FDA = flexible discriminant analysis; RF = random forest; ANN = artificial neural network; MaxEnt = maximum entropy model.


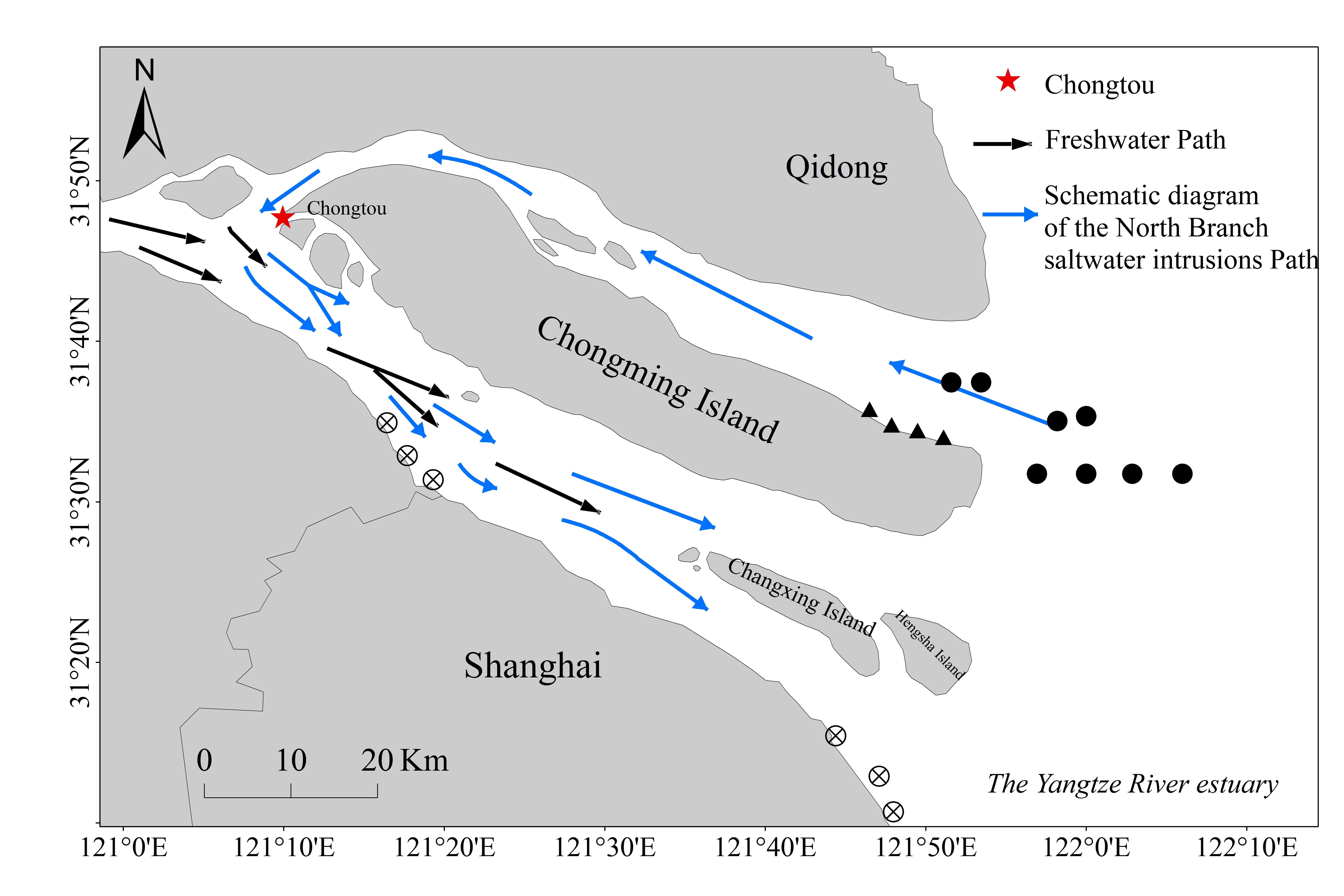


**Fig. A1.** Distribution of *Collichthys lucidus* in the waters of the north and south branches of the Yangtze River Estuary based on stow net and stake-hold net surveys during 1982–1983 (based on the results of Zhang and Zhang (1985))
